# Impact of the Coexisting Coronary Artery Disease on Five-Year Outcomes in Lower Extremity Artery Disease Patients Without Chronic Limb-Threatening Ischemia

**DOI:** 10.7759/cureus.62929

**Published:** 2024-06-22

**Authors:** Eiji Karashima, Keiichiro Kishikawa, Takeshi Arima, Hirotaka Noda, Shioto Yasuda, Takeo Kaneko

**Affiliations:** 1 Cardiology, Shimonoseki City Hospital, Shimonoseki, JPN

**Keywords:** peripheral artery disease (pad), coronary revascularization, coronary artery disease, intermittent claudication, lower extremity artery disease

## Abstract

Coronary artery disease (CAD) is often noted in patients with lower-extremity artery disease (LEAD). However, the effects of CAD on patients with LEAD have not been clearly investigated. In this study, to investigate the effect of CAD on patients with LEAD without chronic limb-threatening ischemia (CLTI), we compared the five-year clinical outcomes of patients with and without CAD. Between 2014 and 2017, 246 patients with symptomatic LEAD without CLTI underwent endovascular treatment. Patients with a history of CAD revascularization or CAD defined by CAD studies were divided into CAD groups, and others were non-CAD groups. After excluding ineligible patients, propensity matching produced 40 patients in each group, and clinical outcomes were compared between the groups. Using five years of Kaplan-Meier analysis between the CAD and non-CAD groups, no significant differences were observed in survival (90.0% vs 92.5%, p=0.693), freedom from cardiovascular events (42.5% vs 57.5%, p=0.110), freedom from LEAD revascularization (67.5% vs 67.5%, p=0.940), and freedom from CLTI (100% vs. 95.0%, p=0.148). However, significant differences were observed in freedom from CAD revascularization (67.5% vs 97.5%, p<0.001) and freedom from symptomatic CAD (85.0% vs 97.5%, p=0.048). Our results suggest that in patients with LEAD without CLTI, CAD caused increased CAD revascularization and symptomatic CAD. However, CAD did not affect survival, cardiovascular events, LEAD revascularization, or CLTI in such patients. When CAD was observed in patients with LEAD without CLTI, more frequent follow-up of CAD may improve the long-term clinical outcomes of such patients.

## Introduction

Due to the aging society and improved diagnostic techniques, the number of patients identified with lower-extremity artery disease (LEAD) is increasing. Endovascular treatment (EVT) is a widely accepted interventional management method for diseased lower extremity arteries. Patients with LEAD have multiple risk factors for atherosclerosis and extensive atherosclerotic disease, significantly increasing their risk of coronary events [1,2]. Coronary artery disease (CAD) is often noted in patients with LEAD. Indeed, the presence of CAD in patients with LEAD was reported to be 25%-70% [1-5], and the presence of CAD in patients with LEAD who undergo EVT was reported to be 43%-56% [6-8]. In addition, CAD is an independent predictor of mortality in patients with LEAD who undergo EVT [9].

Chronic limb-threatening ischemia (CLTI), an advanced form of LEAD, is associated with a poor prognosis [1,2]. The effects of CAD in patients with LEAD and CLTI have been discussed in previous reports [1,2,10,11]. However, the effects of CAD in patients with LEAD without CLTI have not been clearly established. This study aimed to investigate the impact of CAD on the clinical outcomes of patients with LEAD without CLTI.

## Materials and methods

Study design 

This was a retrospective, single-center, and non-randomized study. In this study, LEAD was limited to atherosclerotic disease of the lower-extremity arteries and included the aortoiliac, femoropopliteal, and infrapopliteal arterial segments.

**Figure 1:**
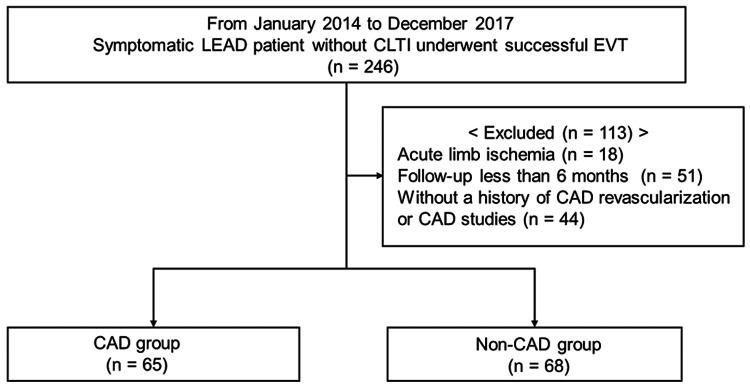
Study population Of the 246 symptomatic LEAD patients without CLTI who underwent successful EVT, 113 were excluded: 18 patients with acute limb ischemia, 51 patients with a follow-up interval of less than six months, and 44 patients without a history of CAD revascularization or CAD studies. Finally, 133 patients were analyzed retrospectively. CAD: coronary artery disease, LEAD: lower-extremity artery disease, CLTI: chronic limb-threatening ischemia, EVT: endovascular treatment

Patients with a history of LEAD revascularization who were admitted to our hospital were excluded from the study. Subsequently, between January 2014 and December 2017, successful EVT was performed in 246 symptomatic patients with LEAD without CLTI at our hospital. Of these, 113 patients were excluded because of acute limb ischemia (18 patients), follow-up of < 6 months (51 patients), and without a history of CAD revascularization or CAD studies (44 patients). Patients were excluded if CAD studies were not performed prior to the first EVT (Figure 1).

Thereafter, 133 patients (mean age, 74.1 ± 7.4 years; 82 male) were analyzed retrospectively. The patients were divided into two groups: the CAD group, 65 patients who had a history of CAD revascularization or CAD detected by CAD studies, and the non-CAD group, 68 patients who did not. Baseline patient characteristics and CAD and LEAD characteristics are summarized in Tables 1, 2, respectively.

**Table 1:** Baseline patient characteristics Values are expressed as mean ± standard deviation or number (percentage). The patients in the CAD group were matched in a 1:1 ratio with those in the non-CAD group based on dyslipidemia, diabetes mellitus, heart failure, ejection fraction < 50%, and hemodialysis. Medication was not included as a factor in matching because medication use differs between the initial visit and after EVT. The nearest-neighbor method with a caliper width of 0.20 on the logit of the propensity score was used for matching. CAD: coronary artery disease, *: significant at p < 0.05, **: significant at p < 0.001.

Variable	All Patients			Propensity Matched		
	CAD (n = 65)	Non-CAD (n = 68)	P-value	CAD (n = 40)	Non-CAD (n = 40)	P-value
Age, years	73.8 ± 7.0	74.4 ± 7.8	0.64	73.1 ± 7.1	74.3 ± 7.8	0.45
Male, n (%)	43 (66.2)	39 (57.4)	0.30	25 (62.5)	23 (57.5)	0.65
Body mass index, (kg/m^2^)	23.1 ± 3.9	21.9 ± 3.1	0.10	22.9 ± 4.4	22.1 ± 3.0	0.30
Hypertension, n (%)	57 (87.7)	54 (79.4)	0.20	34 (85.0)	31 (77.5)	0.39
Dyslipidemia, n (%)	51 (78.5)	37 (54.4)	0.005*	26 (65.0)	28 (70.0)	0.64
Diabetes mellitus, n (%)	40 (61.5)	26 (38.2)	0.007*	21 (52.5)	21 (52.5)	>0.99
Smoking, n (%)	49 (75.4)	49 (72.1)	0.88	31 (77.5)	28 (70.0)	0.56
Cerebrovascular disease, n (%)	23 (35.4)	16 (23.5)	0.14	12 (30.0)	12 (30.0)	>0.99
Atrial fibrillation, n (%)	14 (21.5)	11 (16.2)	0.41	8 (20.0)	9 (22.5)	0.79
Heart failure, n (%)	36 (55.4)	14 (20.6)	<0.001**	14 (35.0)	13 (32.5)	0.82
Ejection fraction < 50%, n (%)	11 (16.9)	2 (2.9)	0.007*	6 (15.0)	2 (5.0)	0.14
Chronic kidney disease, n (%)	37 (56.9)	34 (50.0)	0.43	17 (42.5)	20 (50.0)	0.51
Hemodialysis, n (%)	9 (13.9)	4 (5.9)	0.12	4 (10.0)	4 (10.0)	>0.99
Albumin (g/dL)	4.2 ± 0.4	4.3 ± 0.3	0.13	4.3 ± 0.3	4.3 ± 0.3	0.71
Serum creatinine (mg/dL)	0.94 ± 0.34	0.88 ± 0.27	0.29	0.87 ± 0.27	0.87 ± 0.27	0.99
Estimated glomerular filtration rate	61.6 ± 21.1	61.7 ± 16.8	0.99	61.3 ± 20.2	63.4 ± 18.1	0.62
Low density lipoprotein (mg/dL)	103 ± 36	112 ± 38	0.13	105 ± 29	107 ± 39	0.21
Hemoglobin A1c (%)	6.7 ± 1.1	6.4 ± 1.3	0.13	6.7 ± 1.2	6.6 ± 1.2	0.54
C-reactive protein (mg/dL)	0.22 ± 0.39	0.24 ± 0.41	0.71	0.26 ± 0.49	0.26 ± 0.50	0.91
Medication at initial visit						
Aspirin, n (%)	37 (56.9)	12 (17.7)	<0.001**	19 (47.5)	9 (22.5)	0.020*
Thienopyridine, n (%)	32 (49.2)	22 (32.4)	0.048*	20 (50.0)	13 (32.5)	0.11
Cilostazol, n (%)	8 (12.3)	15 (22.1)	0.14	5 (12.5)	9 (22.5)	0.25
Oral anticoagulants, n (%)	5 (7.7)	6 (8.8)	0.81	2 (5.0)	5 (12.5)	0.24
Statin, n (%)	33 (50.8)	19 (27.9)	0.007*	18 (45.0)	15 (37.5)	0.50
Insulin, n (%)	13 (20.0)	5 (7.4)	0.034*	5 (12.5)	4 (10.0)	0.73
Medication after EVT						
Aspirin, n (%)	48 (73.9)	18 (26.5)	<0.001**	29 (72.5)	15 (37.5)	0.001*
Thienopyridine, n (%)	52 (80.0)	44 (64.7)	0.05	33 (82.5)	23 (57.5)	0.014*
Cilostazol, n (%)	16 (24.6)	43 (63.2)	<0.001**	12 (30.0)	25 (62.5)	0.003*
Oral anticoagulants, n (%)	9 (13.9)	7 (10.3)	0.53	5 (12.5)	6 (15.0)	0.75
Statin, n (%)	51 (78.5)	49 (72.1)	0.40	29 (72.5)	34 (85.0)	0.18
Insulin, n (%)	14 (21.5)	5 (7.4)	0.019*	6 (15.0)	4 (10.0)	0.51

**Table 2:** CAD and LEAD characteristics Values are expressed as numbers (percentage). CAD: coronary artery disease, EVT: endovascular treatment, LEAD: lower extremity artery disease, *: significant at p < 0.05, **: significant at p < 0.001.

Variable	All patients			Propensity matched		
	CAD (n = 65)	Non-CAD (n = 68)	P-value	CAD (n = 40)	Non-CAD (n = 40)	P-value
History of CAD						
History of CAD revascularization, n (%)	21 (32.3)	0 (0.0)	<0.001**	9 (22.5)	0 (0.0)	<0.001**
History of acute myocardial infarction, n (%)	9 (13.8)	0 (0.0)	0.001*	4 (10.0)	0 (0.0)	0.041*
Planned CAD revascularization after CAD studies, n (%)	42 (64.6)	0 (0.0)	<0.001**	26 (65.0)	0 (0.0)	<0.001**
CAD studies						
Treadmill tests, n (%)	2 (3.1)	3 (4.4)	0.61	0 (0.0)	1 (2.5)	0.32
Coronary computed tomography angiography, n (%)	10 (15.4)	8 (11.8)	0.69	6 (15.0)	4 (10.0)	0.51
Coronary angiography, n (%)	59 (90.8)	65 (95.6)	0.17	36 (90.0)	38 (95.0)	0.40
Myocardial scintigraphy, n (%)	5 (7.7)	3 (4.4)	0.51	2 (5.0)	1 (2.5)	0.46
Rutherford classification						
Category 2, n (%)	38 (58.5)	22 (32.4)	0.010*	20 (50.0)	24 (60.0)	0.38
Category 3, n (%)	27 (41.5)	46 (67.6)	0.010*	20 (50.0)	16 (40.0)	0.38
Ankle brachial index						
Pre EVT	0.66 ± 0.15	0.68 ± 0.18	0.42	0.64 ± 0.15	0.66 ± 0.17	0.70
Post EVT	0.82 ± 0.15	0.87 ± 0.15	0.06	0.83 ± 0.15	0.88 ± 0.15	0.19
Chronic total occlusion segments of lower extremity arteries						
Aortoiliac, n (%)	13 (20.0)	10 (14.7)	0.42	11 (27.5)	6 (15.0)	0.18
Femoropopliteal, n (%)	18 (27.7)	20 (29.4)	0.83	12 (30.0)	12 (30.0)	>0.99
Segments of EVT						
Aortoiliac, n (%)	39 (60.0)	36 (52.9)	0.42	27 (67.5)	20 (50.0)	0.12
Femoropopliteal, n (%)	40 (61.5)	42 (61.8)	0.98	21 (52.5)	25 (62.5)	0.37
Infrapopliteal, n (%)	1 (1.5)	0 (0.0)	0.31	1 (2.5)	0 (0.0)	0.32

Detailed information and baseline clinical data were collected on admission. Clinical evaluations were performed at one, six, and 12 months after the first EVT and every six months thereafter. When revascularization of the CAD or LEAD was planned at the time of the first EVT, those revascularizations were not enrolled as events of CAD or LEAD revascularization. The study protocol was developed in accordance with the Declaration of Helsinki and approved by the hospital’s ethics committee (number 2023SCHEC-012). Written informed consent was obtained from all the study patients.

Definitions

The follow-up period was defined as five years after the first EVT. Chronic kidney disease was defined as an estimated glomerular filtration rate less than 60 mL/min/1.73m². CAD studies included treadmill tests, coronary computed tomography angiography, coronary angiography, and myocardial scintigraphy. Cardiovascular events included cardiovascular death, CAD revascularization, symptomatic CAD, LEAD revascularization, CLTI, and cerebral infarction. Symptomatic CAD was defined as acute myocardial infarction or angina with CAD symptoms.

Statistical analysis

Continuous data are presented as mean ± standard deviation, and categorical data are presented as numbers (percentages). Continuous variables were compared between groups using an unpaired t-test or Mann-Whitney U test, as appropriate. Categorical data were compared using the Chi-square test. The patients in the CAD group were matched in a 1:1 ratio with those in the non-CAD group based on dyslipidemia, diabetes mellitus, heart failure, ejection fraction < 50%, and hemodialysis. Medication was not included as a factor in matching because medication use differs between the initial visit and after EVT. The nearest-neighbor method with a caliper width of 0.20 on the logit of the propensity score was used for matching. Time-dependent outcomes were analyzed using the Kaplan-Meier method, and curves were compared using the log-rank test. Statistical significance was set at p < 0.05. All the statistical analyses were performed using SPSS version 27 (IBM Corp., Armonk, NY, USA).

## Results

Of the 133 patients (mean age 74.1±7.4; 82 men) in this study, 65 (48.9%) were classified into the CAD group. Propensity matching produced 40 patients (mean age 73.1±7.1; 25 men) with CAD and 40 patients (mean age 74.3±7.8; 23 men) without CAD. Table 1 summarizes the baseline patient characteristics of the unmatched and matched populations. In the unmatched samples of the baseline characteristics, significant between group differences were observed in dyslipidemia, diabetes mellitus, heart failure, and ejection fraction < 50%, aspirin, thienopyridine, cilostazol, statin, and insulin. Antiplatelet use increased after EVT compared to the initial visit. Aspirin use was more common in the CAD group and cilostazol use was more common in the non-CAD group. In both groups, statin use clearly increased after EVT compared to the initial visit. Among the CAD and LEAD characteristics shown in Table 2, history of CAD revascularization, history of acute myocardial infarction, and planned CAD revascularization after CAD studies were only shown in the CAD group. There was no significant difference between CAD and non-CAD groups in the CAD studies. As a CAD study, coronary angiography was most frequently performed in this study. The rate of Rutherford category 2 was higher in the CAD group. However, the distribution of chronic total occlusion and EVT segments that were performed to improve LEAD symptoms on admission did not differ between the groups. After propensity score matching, except for medication, most baseline and LEAD characteristics were well-balanced between the groups.

**Figure 2:**
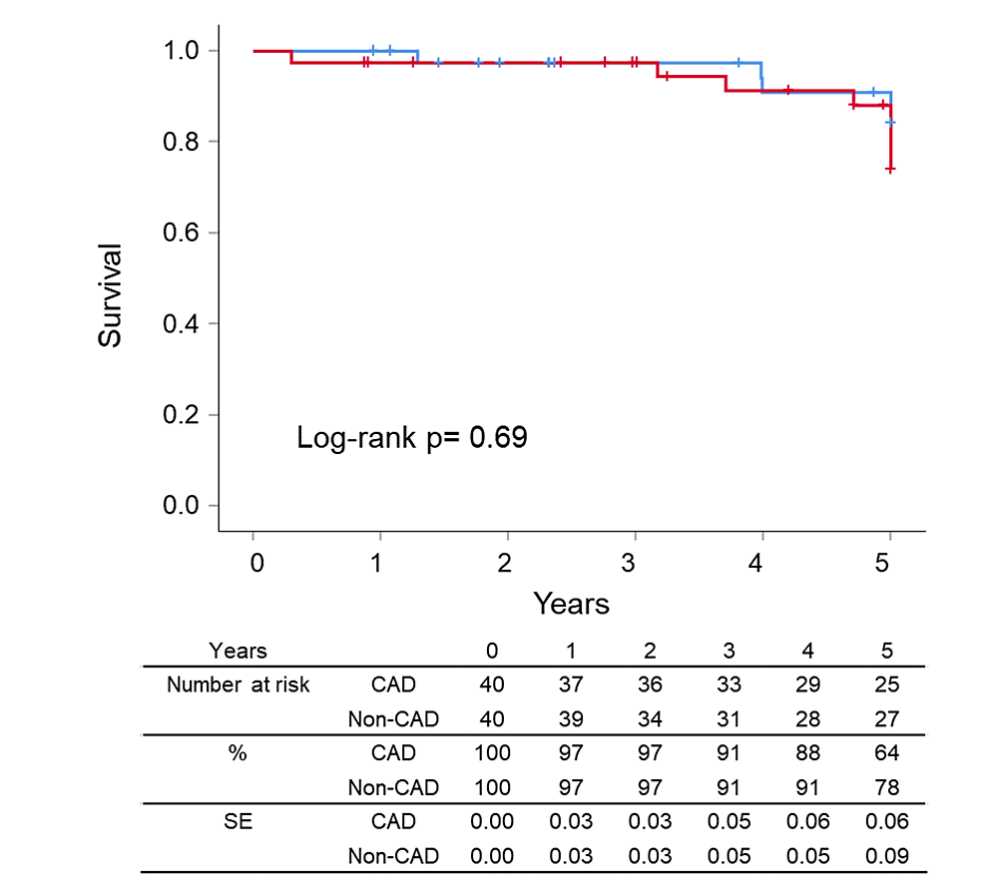
Kaplan–Meier curves for survival CAD: coronary artery disease, SE: standard error

**Figure 3:**
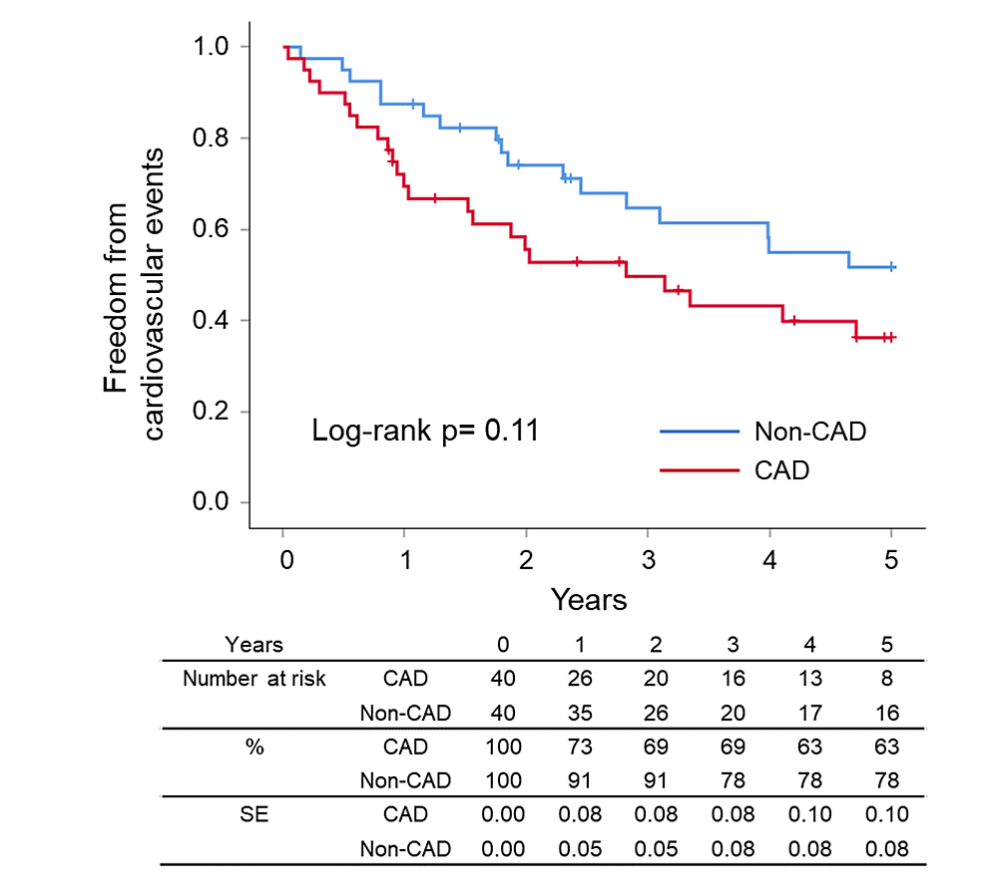
Kaplan–Meier curves for freedom from cardiovascular events Cardiovascular events included cardiovascular death, acute myocardial infarction or angina with CAD symptoms, CAD revascularization, revascularization of lower extremity artery disease, chronic limb-threatening ischemia, and cerebral infarction. CAD: coronary artery disease, SE: standard error

**Figure 4:**
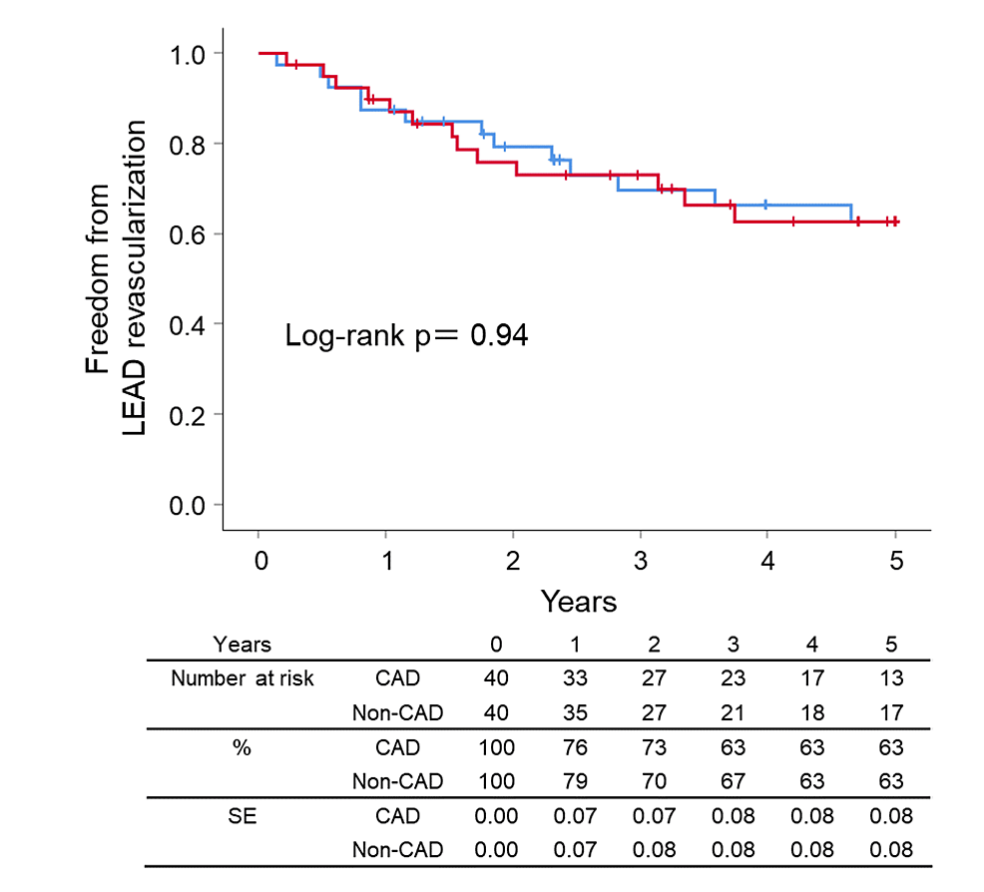
Kaplan–Meier curves for freedom from LEAD revascularization CAD: coronary artery disease, LEAD: lower extremity artery disease, SE: standard error

**Figure 5:**
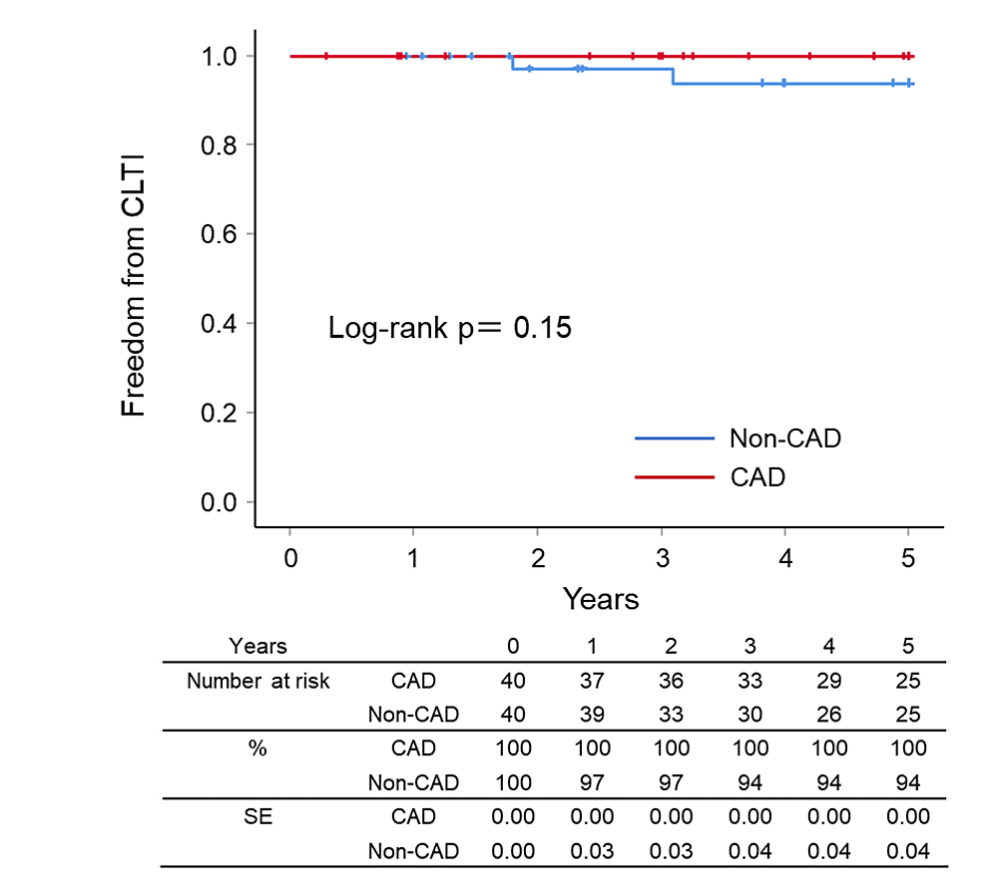
Kaplan–Meier curves for freedom from CLTI CAD: coronary artery disease, CLTI: chronic limb-threatening ischemia, SE: standard error

**Figure 6:**
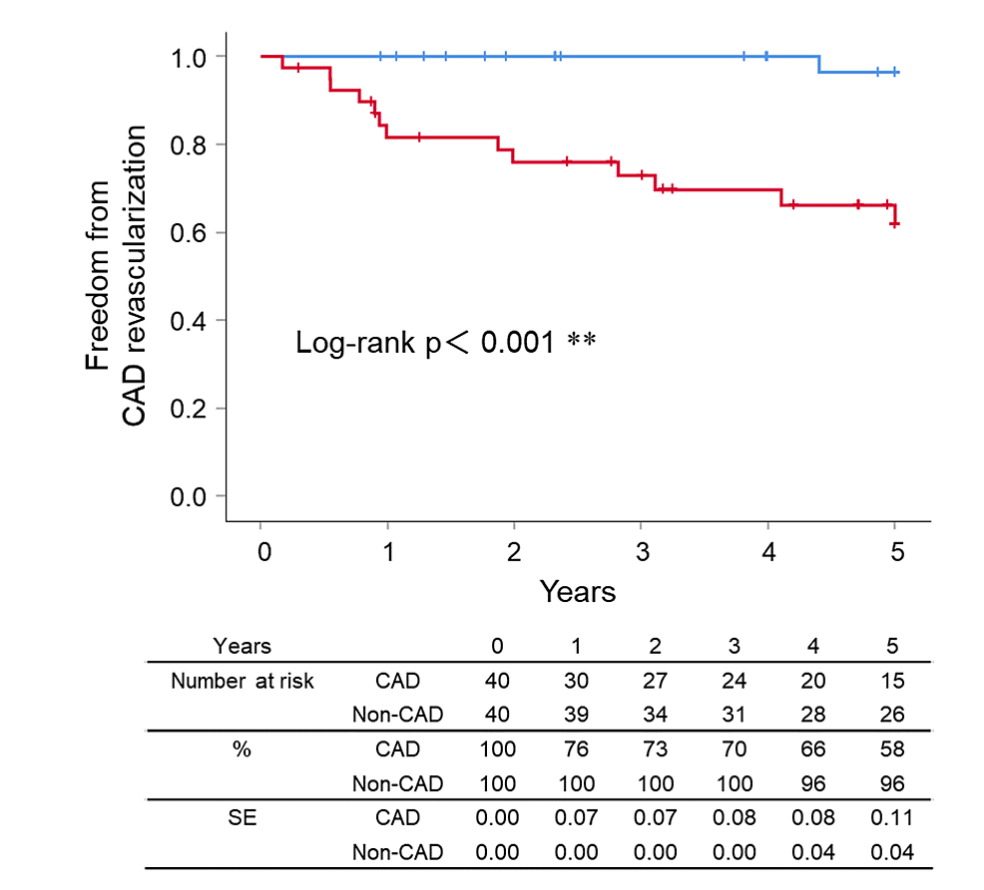
Kaplan–Meier curves for freedom from CAD revascularization CAD: coronary artery disease, SE: standard error, **: significant at p < 0.001

**Figure 7:**
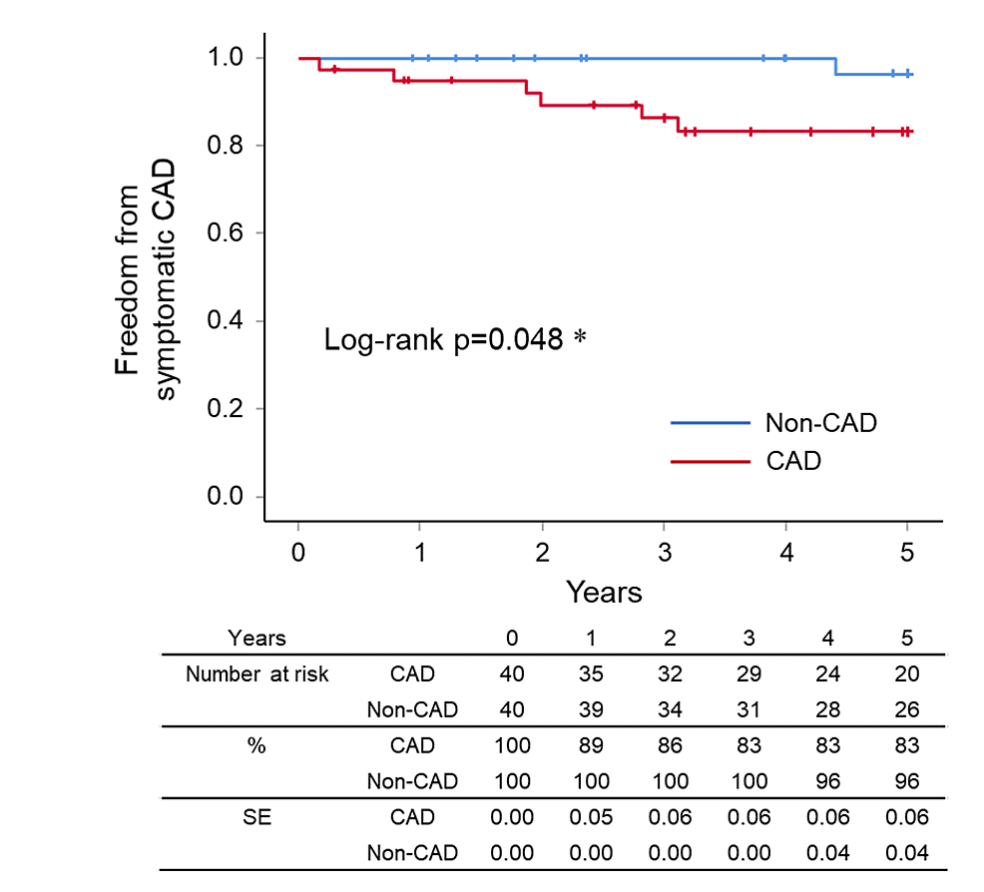
Kaplan–Meier curves for freedom from symptomatic CAD Symptomatic CAD was defined as the acute myocardial infarction or angina with CAD symptoms. CAD: coronary artery disease, SE: standard error, *: significant at p < 0.05

After the successful EVT, the ankle-brachial index value rose from 0.65±0.16 to 0.86±0.15 in the matched patients. Clinical outcomes such as survival, cardiovascular events, LEAD revascularization, CLTI, CAD revascularization, and symptomatic CAD were summarized in Figures 2-7, respectively. At five years, the Kaplan-Meier analysis shows that there was no significant difference between CAD and non-CAD groups in the survival (90.0% (95% CI 85.1% to 94.9%) vs 92.5% (95% CI 88.6% to 96.4%), p=0.693) (Figure 2), freedom from cardiovascular events (42.5% (95% CI 33.4% to 51.6%) vs 57.5% (95% CI 48.8% to 66.2%), p=0.110) (Figure 3), and freedom from LEAD revascularization (67.5% (95% CI 56.5% to 77.3%) vs 67.5% (95% CI 58.1% to 76.9%), p=0.940) (Figure 4).

During the follow-up period, CLTI was not observed in the CAD group but was observed in two patients in the non-CAD group. Therefore, the Kaplan-Meier analysis showed no significant difference between the groups in the freedom from CLTI (100% vs. 95.0%, p=0.148) (Figure 5).

However, the Kaplan-Meier analysis showed a significant difference between the groups in the freedom from CAD revascularization (CAD vs non-CAD; 67.5% (95% CI 57.5% to 77.5%) vs 97.5% (95% CI 96.7% to 98.3%), p<0.001) (Figure 6) and freedom from symptomatic CAD (CAD vs non-CAD; 85.0% (95% CI 77.4% to 92.6%) vs 97.5% (95% CI 96.7% to 98.3%), p=0.048) (Figure 7) at five years.

## Discussion

Compared with LEAD patients with CLTI, the clinical outcomes of LEAD patients without CLTI are favorable [1,2]. Therefore, the effects of CAD have not been clearly investigated in patients with LEAD without CLTI. Our study found that when CAD coexisted in patients with LEAD without CLTI, the incidents of CAD revascularization and symptomatic CAD were significantly increased during the five-year follow-up period.

The risk of LEAD increases with exposure to major cardiovascular risk factors including smoking, hypertension, dyslipidemia, and diabetes [1,2]. Cumulative exposure to these cardiovascular risk factors increases the incidence of cardiovascular diseases [12]. As shown in Table 1, the prevalence rates of major cardiovascular risk factors in the unmatched population were higher in the CAD group than in the non-CAD group. However, the duration of major cardiovascular risk factors in this study was not investigated. Therefore, we cannot exclude the possibility that the duration of major cardiovascular risk factors was longer in patients with CAD than in those without.

Performing CAD studies of all patients undergoing EVT are unrealistic. In addition, it is sometimes difficult to investigate CAD in patients with LEAD because they are often asymptomatic. Moreover, the definition of CAD may have affected the results of our study. All patients in this study underwent some type of investigation for CAD or had a history of coronary revascularization. The difference in sensitivities of CAD studies for ischemia might affect the diagnosis of CAD in the present group. The sensitivity of the treadmill exercise test could be lower in patients with LEAD because of limited physical activity, it might not be appropriate for the present study group.

Compared to the patients with CAD alone, patients with combined CAD and LEAD have significantly worse clinical outcomes [1-3,13,14]. In addition, patients with combined CAD and LEAD have a high risk of cardiovascular death [15]. It has also been reported that in patients with combined CAD and LEAD, clinical outcomes were significantly better with complete CAD revascularization than with incomplete CAD revascularization [16]. Moreover, compared to patients with CAD, the control of cardiovascular risk factors is poor in patients with LEAD [17]. Considering these findings, the recognition of CAD in patients with LEAD may improve the risk factor control and long-term clinical outcomes of such patients.

Only one randomized controlled trial was designed to assess the impact of systematic screening for multisite artery disease on prognosis [1,18]. In that study, LEAD screening in patients with CAD failed to prove any significant benefit [18]. However, the population of that study was limited to patients with CAD and the benefit of CAD screening in patients with LEAD was not investigated. The coexistence rate of CAD and LEAD is quite different between the patients with CAD and LEAD. Indeed, the presence of CAD in patients with LEAD was reported to be 25%-70% [1-5], and the presence of CAD in patients with LEAD who undergo EVT was reported to be 43%-56% [6-8]. Conversely, less frequency of LEAD in CAD patients has been reported to be 7%-16% [1,2,14,15]. The real benefits of CAD screening in patients with LEAD need to be determined in future randomized controlled trials.

In this study, CAD studies investigated CAD in nearly half of LEAD patients without CLTI. After the CAD studies, planned CAD revascularizations were performed in more than 60 % of patients in the CAD group. In addition, compared to the non-CAD group, the incidents of CAD revascularization and symptomatic CAD were increased in the CAD group during the five-year follow-up period. These findings suggest that it may be useful to perform CAD studies in LEAD patients without CLTI.

Limitations

This study had some limitations. This was a non-randomized, retrospective, single-center study with a small number of patients, and selection bias could not be eliminated. The number of cases was too small and there was insufficient power to detect an effect on mortality and cardiovascular events. Another limitation is that about half of the patients enrolled (113 out of 246) were excluded. Treatment strategies for symptomatic LEAD depend on the surgeon’s discretion. In addition, we did not discuss the CAD treatment strategies. Finally, this study lacked an independent core angiography laboratory.

## Conclusions

In patients with LEAD without CLTI, the five-year clinical outcomes of our study indicated that the coexistence of CAD caused increased CAD revascularization and symptomatic CAD. However, the coexistence of CAD in such patients did not affect survival, cardiovascular events, LEAD revascularization, or CLTI in this study. Randomized studies with a larger number of patients are needed to confirm our preliminary results.
